# Response to the Letter to the Editor Entitled “A World Without Dialysis or a World With Dialysis for All Who Need It”

**DOI:** 10.1016/j.ekir.2025.09.038

**Published:** 2025-10-08

**Authors:** Navdeep Tangri, Stacey Priest, Bo Ren Long, Jieling Chen, Naveen Rao, Clélia-Elsa Froguel, Breonny Robson, Nick Guldemond, Ana Flavia Moura, Ralph Audehm, Fiona Adshead, Ming-hui Zhao, Steven Chadban

**Affiliations:** 1 University of Manitoba, Winnipeg, Manitoba, Canada; 2 Value & Evidence, EVERSANA, Burlington, Ontario, Canada; 3 BioPharmaceuticals Medical, AstraZeneca, Gaithersburg, Maryland, USA; 4 BioPharmaceuticals Medical, AstraZeneca, Cambridge, UK; 5 Kidney Health Australia, Melbourne, Victoria, Australia; 6 Leiden University Medical Centre, Leiden, Netherlands; 7 Escola Bahiana de Medicina e Saúde Pública, Salvador, Brazil; 8 Department of General Practice, University of Melbourne, Melbourne, Victoria, Australia; 9 Sustainable Healthcare Coalition, Devon, UK; 10 Renal Division, Peking University First Hospital Peking University Institute of Nephrology Beijing, PR China; 11 Royal Prince Alfred Hospital, Camperdown, New South Wales, Australia

The Author Replies

We appreciate the insightful letter to the editor by Torreggiani and Piccoli^1^ highlighting the importance of kidney care and dialysis in response to our original article outlining the impact of improved diagnosis and guideline-directed medical therapy on holistic chronic kidney disease (CKD) burden.^2^

We align with Torreggiani and Piccoli’s^1^ view that dialysis should be accessible to all who need it, and that sustained investment in kidney health services is essential. Determining how investment in kidney care might be most effectively directed between options, including screening and diagnosis, optimal pharmacotherapy, kidney replacement therapy, or health care infrastructure, was outside the scope of our paper. Instead, our findings highlight that prioritizing timely diagnosis and treatment can reduce CKD burden and enhance health care system capacity. Alongside the 75% guideline-directed medical therapy adherence scenario presented in the main body of our manuscript, we included sensitivity analyses to allow for plausible variations in health behaviors and determinants of outcomes, and these supported our conclusions.^2^

Our modelling found that improved diagnosis and treatment of CKD would lead to increased life expectancy within the CKD population and would reduce progression to kidney failure.^2^ The net effect is a modest decline in dialysis requirements (accompanied by modest reductions in total kidney replacement therapy costs) over a 10-year timeframe, compared with substantial increases if we fail to improve diagnosis and treatment. We note that relationships between mortality, kidney replacement therapy, and treatment are dynamic and are modelled as such in our study. One-way scenario analysis as suggested in the letter would not be suitable for illustrating these complex dynamics.

A core goal of CKD care is to improve life expectancy and quality of life, while minimizing kidney replacement therapy growth. We modelled better detection and implementation of guideline-directed medical therapy. As noted as a limitation in our discussion, there are other approaches to achieving this goal, such as innovation in CKD therapy and changes in guidelines.^2^ Incorporating these effects would likely amplify the projected benefits.

We endorse health policies that prioritize early CKD detection and treatment to slow progression, improve patient outcomes, reduce productivity losses, and alleviate pressure on health care systems by decreasing dialysis demand.^3^
